# α-Tocopherol at Nanomolar Concentration Protects Cortical Neurons against Oxidative Stress

**DOI:** 10.3390/ijms18010216

**Published:** 2017-01-21

**Authors:** Irina O. Zakharova, Tatiana V. Sokolova, Yulia A. Vlasova, Liubov V. Bayunova, Maria P. Rychkova, Natalia F. Avrova

**Affiliations:** 1 Department of Molecular Endocrinology and Neurochemistry, I.M. Sechenov Institute of Evolutionary Physiology and Biochemistry of the Russian Academy of Sciences, Thorez avenue, 44, Saint-Petersburg 194223, Russia; zakhar@iephb.ru (I.O.Z.); sokolt1956@mail.ru (T.V.S.); yousia@mail.ru (Y.A.V.); bayunoval@mail.ru (L.V.B.); involved@mail.ru (M.P.R.); 2 Preventive Medicine Department, Mechnikov North-West StateMedical University, Saint-Petersburg, Kirochnaya ul. 41, Saint-Petersburg 191015, Russia

**Keywords:** brain cortical neurons, H_2_O_2_, α-tocopherol, nanomolar and micromolar concentrations, viability, reactive oxygen species, ERK1/2, Akt, protein kinase Cδ, Bax/Bcl-2 ratio

## Abstract

The aim of the present work is to study the mechanism of the α-tocopherol (α-T) protective action at nanomolar and micromolar concentrations against H_2_O_2_-induced brain cortical neuron death. The mechanism of α-T action on neurons at its nanomolar concentrations characteristic for brain extracellular space has not been practically studied yet. Preincubation with nanomolar and micromolar α-T for 18 h was found to increase the viability of cortical neurons exposed to H_2_O_2_; α-T effect was concentration-dependent in the nanomolar range. However, preincubation with nanomolar α-T for 30 min was not effective. Nanomolar and micromolar α-T decreased the reactive oxygen species accumulation induced in cortical neurons by the prooxidant. Using immunoblotting it was shown that preincubation with α-T at nanomolar and micromolar concentrations for 18 h prevented Akt inactivation and decreased PKCδ activation induced in cortical neurons by H_2_O_2_. α-T prevented the ERK1/2 sustained activation during 24 h caused by H_2_O_2_. α-T at nanomolar and micromolar concentrations prevented a great increase of the proapoptotic to antiapoptotic proteins (Bax/Bcl-2) ratio, elicited by neuron exposure to H_2_O_2_. The similar neuron protection mechanism by nanomolar and micromolar α-T suggests that a “more is better” approach to patients’ supplementation with vitamin E or α-T is not reasonable.

## 1. Introduction

The development of oxidative stress is one of the main causes of brain nerve cell damage and death in neurodegenerative and ischemic diseases, such as Parkinson’s and Alzheimer’s diseases or brain insult. These widespread diseases result in cognitive dysfunction, disablement and death for elderly people. For many years it was hoped that vitamin E might be used as a remedy for various diseases concerned with pathological accumulation of reactive oxygen species (ROS) in the cells of various organs. However, analysis of the results of clinical trials revealed the unfavorable effect of administering high doses of vitamin E. Thus, analysis of the published results of randomized clinical trials (more than 100,000 observations) of vitamin E administration to people with various diseases showed that the all-cause mortality for patients and people in risk groups who received high doses of vitamin E in their diet was higher than for those who received a placebo [1,2,3]. The main and most active component of vitamin E in various organs in humans and animals is α-tocopherol (α-T). Specific α-T transfer and binding proteins prevent it from metabolic degradation. The above-mentioned clinical trial results show the need for studying the mechanism of action of α-T and vitamin E other components at their physiological concentrations, at which they exert their effects on the cells of various organs and on various cells of the same organ in vivo, and which may differ from their effect at high pharmacological concentrations [4]. Thus, it was shown that ischemic stroke-induced brain injury was exacerbated in the presence of supraphysiologic brain α-T level [4].

The delivery of vitamin E components from the blood to the cerebrospinal fluid takes place across the blood-brain barrier. α-T is the main vitamin E component. In cerebrospinal fluid its concentration was found to be 42.1 ± 17 nM, while the concentration of other vitamin E components was much lower—for example, the γ-tocopherol concentration was found to be 5.9 ± 2.8 nM [5]. Similar data were obtained in other studies (see, for example, [6]).

Various vitamin E isoforms (α-, β-, γ- and δ-tocopherols and tocotrienols) not only possess a radical scavenging activity, but also modulate the activity of a large number of signaling pathways; in most cases the data were originally obtained using non-neural cells. Thus, α-T was shown to inhibit the activity of the protein kinase C (PKC), the extracellular signal-regulated 1/2 protein kinase (ERK1/2) and the phosphatidylinositol 3-kinase (PI 3-kinase)/Akt pathway, and to activate protein phosphatases, especially protein phosphatase 2A and lipid phosphatase, e.g., dual specificity phospatase PTEN (phosphatase and tensin homologue), as well as to modulate the activity of cyclooxygenase 1/2, lipoxygenases, NADPH oxidase and the function of ion channels [6,7,8,9]. In contrast to “non-neural” cells, α-T was found to activate basal ERK1/2 and Akt in brain cortical neurons [10]. These effects of α-T are sometimes called the “non-antioxidant functions of α-T”, though the signaling pathways modulation may lead to a decrease in the ROS accumulation in cells. Various components of vitamin E differ from one another much more in their modulation of signal transduction pathways than in the level of their scavenging activity [7,8].

The majority of studies of the α-T protective effect on nerve cells is performed using micromolar concentrations. At the same time, nanomolar α-tocotrienol was found to increase the viability of both hippocampal neurons and cells of the HT4 hippocampal cell line exposed to glutamate; its effect was shown to depend on the inhibition of 12-lipoxygenase and phospholipase A_2_ [11,12]. Data on the protective effect of α-T at nanomolar concentrations on nerve cells and cells of neuronal cell lines are not abundant [10,13,14]. The protective effect of 250 nM α-T against 10 mM glutamate-induced death of immature brain cortical neurons was shown to be much less pronounced than the protective effect of 2.5 µM α-T [13]. It was also shown [10,14] that long preincubation (18–24 h) with α-T at nanomolar concentrations increased its protective effect in rat brain cortical neurons and PC12 cells. The protective effect of nanomolar α-T against the H_2_O_2_-induced death of brain cortical neurons [10] or PC12 cells [14] was comparable with the protective effect of micromolar α-T after preincubation for 18–24 h.

According to Numakawa and co-authors, α-T at nanomolar concentrations was shown to increase the activity of basal ERK1/2 and protein kinase B (Akt), as well as the Bcl-2 level in cortical neurons [10]. However, neither this nor other studies have shown how H_2_O_2_ modulates the activity of ERK1/2 and Akt, and how α-T diminishes or abolishes the toxic effects of H_2_O_2_ in the nerve cells.

The aim of the present work is to study the effect of H_2_O_2_ and long (18 h) preincubation with α-T at nanomolar and micromolar concentrations on the viability of immature rat brain cortical neurons in culture, on the activity of protein kinases and the ratio of pro- to antiapoptotic proteins in these neurons. It was found that long (18 h) preincubation of cortical neurons with α-T at nanomolar or micromolar concentrations prevented the inactivation of Akt and pronounced decrease of Bcl-2 level leading to an increase of the proapoptotic to antiapoptotic protein ratio (Bax/Bcl-2) in neurons induced by cell exposure to H_2_O_2_. Nanomolar and micromolar α-T also diminished the activation of PKCδ induced by H_2_O_2_ and the time of maximal activation of ERK1/2 by this prooxidant in brain cortical neurons.

## 2. Results and Discussion

### 2.1. α-T and the Viability and Function of Nerve Cells

A vitamin E or α-T deficiency leads to an abnormal functioning of the brain. Thus, mutations in the α-T transfer protein gene result in a disease called “ataxia with vitamin E deficiency” [15], vitamin E supplementation to such patients’ diet gives good results [15]. However, administration of vitamin E to patients with neurodegenerative diseases was not helpful. It was even suggested that it might be “time to stop feeding vitamin E to dementia patients” [16]. There are data showing that the mechanism of α-T effect on nerve cells metabolism, viability and functioning is rather complicated; and appears not to be exclusively limited to its scavenging activity. Thus, the long-term (up to 46 weeks) maintenance of mice on a diet with a deficit of vitamin E and the use of mice with targeted disruption of the α-T transfer protein gene (which mutations lead to the above-mentioned hereditary ataxia with vitamin E deficiency in humans) resulted in a large decrease of the α-T content in the brain and other organs [17]. However, the intensity of the lipid peroxidation processes in the brain of these mice was not increased; on the contrary, it was markedly diminished, and exposure of the brain tissue to α-T greatly increased ROS production and accumulation [17].

The long pre-treatment of hippocampal neurons with 1–2.5 µM α-T prior to the induction of oxidative stress provided a long-lasting protection via genomic activation, which was in contrast with the transient effect of α-T on neuron viability based on its radical scavenging activity [18,19].

The effects of α-T and other vitamin E components at their physiological nanomolar concentrations [5,6] characteristic for cerebrospinal fluid and brain extracellular space (at which they act on various brain cells in vivo) may differ from their effects at much higher pharmacological concentrations [4,7], especially in the case of long-term administration to humans or animals. Thus, it was shown that excessive α-T in the brain exacerbated microglial activation and brain injury caused by acute ischemic stroke in mice [4].

### 2.2. The Dosage and Duration of Treatment with α-T Govern Its Neuroprotective Effect

**Figure 1:**
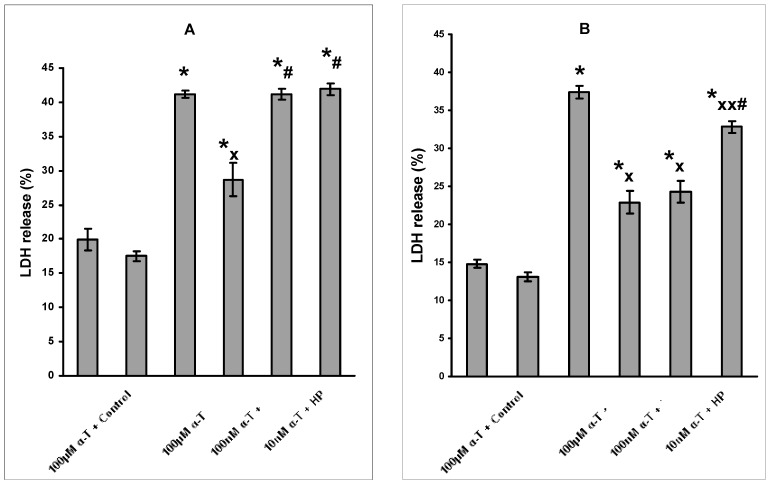
Shows that preincubation of immature brain cortical neurons with α-tocopherol (α-T) at nanomolar concentrations for 0.5 h prior to exposure of the cells to 0.2 mM H_2_O_2_ for 24 h did not increase the viability of brain cortical neurons (**A**); while preincubation with 10 and 100 nM α-T for 18 h prior to exposure of the neurons to 0.2 mM H_2_O_2_ for 24 h caused a pronounced increase in cortical neuron viability (**B**). H_2_O_2_ is designated as HP in the figure. Lactate dehydrogenase (LDH) method was used to determine neuron viability. The data bars (from the left to the right) in (**A**,**B**) show: (1) control values of LDH release from neurons; (2) control values of LDH release after incubation of the neurons with 100 µM α-T for 30 min (**A**) or for 18 h (**B**); (3) LDH release after neuron exposure to H_2_O_2_; (4) LDH release after preincubation of the neurons with 100 µM α-T prior to the cell exposure to H_2_O_2_; (5) LDH release after preincubation of the neurons with 100 nM α-T prior to the cell exposure to H_2_O_2_; (6) LDH release after preincubation of the neurons with 10 nM α-T prior to the cell exposure to H_2_O_2_. The results of one typical experiment from 9 experiments performed are shown as means ± SEM from 2–3 determinations in parallel samples. One way ANOVA followed by Tukey’s multiple comparison test was used to assess the significance of the differences between various groups of data. The differences were found to be significant: *—compared to control values, *p* < 0.01; ^x^ and ^xx^—compared to the effect of H_2_O_2_ alone; ^x^ *p* < 0.01, ^xx^ *p* < 0.05, ^#^—compared to the effect of α-T higher concentrations, *p* < 0.01.

If neurons were preincubated with α-T for 18 h and then exposed to 0.2 mM H_2_O_2_, the protective effect of α-T at 100 nM and 100 µM concentrations on neurons in complete incubation medium containing serum was similar and significant (Figure 1B). Preincubation with 10 nM α-T for 18 h also increased viability of brain cortical neurons, but to a lesser extent than preincubation with α-T at 100 nM and higher concentration (Figure 1B). The data obtained in the experiments were expressed as α-T rescue rates (Table 1).

We also studied the correlation between α-T concentrations (using a logarithmic scale) and its rescue rates at these concentrations. The most well-defined positive correlation (*n* = 28, *r*^2^ = 0.53, *r* = 0.728, *p* < 0.0001) between these two variables was achieved when the effects of 10^−9^, 10^−8^ and 10^−7^ M α-T (1, 10 and 100 nM α-T) were compared. A less pronounced positive correlation between these two variables (*n* = 53, *r*^2^ = 0.389, *r* = 0.62, *p* < 0.001) was revealed when comparing the rescue rates characteristic for all the concentrations studied and presented in Table 1. There is no correlation at all between α-T concentrations of 10^−7^ M and higher and α-T rescue rates at these concentrations (*n* = 36, *r*^2^ = 0.04, *r* = 0.19, *p* = 0.26). This can clearly be seen on the logarithmic scale graphs.

The data presented in Table 1 as well as the data about correlation between α-T concentrations and its rescue rates at these concentrations provide evidence that the protective effect of α-T on brain cortical neurons against H_2_O_2_-induced death was concentration-dependent in the range 1–100 nM (1 nM < 10 nM < 100 nM) if preincubation was performed for 18 h. Preincubation with 1 nM α-T did not significantly protect brain cortical neurons against H_2_O_2_-induced toxicity; the rescue rate of 10 nM α-T was significantly higher than that of 1 nM α-T and its protective effect was significant. The α-T rescue rates at 100 nM, 1 µM, 10 µM and 100 µM concentrations did not significantly differ from one another, but were higher than the rescue rate of α-T at 10 nM concentration (Table 1).

Previously, we have shown [14] that the protective effect of α-T against H_2_O_2_-induced PC12 cell death was also higher the higher was the α-T concentration in the nanomolar range (1 nM < 10 nM < 100 nM). Numakawa and co-authors were the first to show the protective effect of nanomolar α-T, but did not reveal its dependence on the α-T concentration [10].

Another piece of evidence of the protective role that preincubation of neuronal cells with α-T at nanomolar concentrations plays was provided by studies of α-T ability to increase viability of PC12 cells exposed to eleostaric acid, which caused increase in ROS production and apoptotic cell death [20].

### 2.3. The Protective Effect of Preincubation with α-T for 18 h against H_2_O_2_-Induced Death of Brain Cortical Neurons Is Diminished or Abolished in the Presence of Inhibitors of PI 3-Kinase, MEK1/2 and PKCδ

The data obtained are presented in Table 2.

The rescue rates were calculated either in the absence of the inhibitor in all samples or in the presence of the same inhibitor in all samples. In most—but not in all—experiments, the LDH release was higher in presence of both H_2_O_2_ and one of the inhibitors than in presence of H_2_O_2_ alone, but the difference was not significant. If the effect of H_2_O_2_ alone is taken for 100%, the combined effect of H_2_O_2_ and LY294002, SL327 or rottlerin is respectively 122.5% ± 10.1%, 115% ± 9.6% or 116% ± 7% (*p >* 0.05).

In presence of the MEK1/2/ERK1/2 and the PI 3-kinase/Akt pathways inhibitors and of the PKCδ inhibitor, the ability of 100 nM and 100 µM α-T to rescue neurons from H_2_O_2_-induced death was markedly and significantly diminished or became insignificant (Table 2).

However, such data should be interpreted with caution. For example, rottlerin was shown not to be a specific inhibitor of PKCδ; it inhibits or activates other metabolic processes in cells [21,22], including the modulation of some protein kinase activity, for example, Ca^2+^/calmodulin-dependent protein kinase activity.

The protective effect of α-T at nanomolar and micromolar concentrations against H_2_O_2_-induced PC12 cell death [14] was also shown to be markedly diminished in the presence of PI 3-kinase and MEK1/2 inhibitors if the cells were preincubated with α-T for 18 h.

### 2.4. α-T at Micromolar and Nanomolar Concentrations Diminishes the Accumulation of ROS Induced in Brain Cortical Neurons by H_2_O_2_

**Figure 2:**
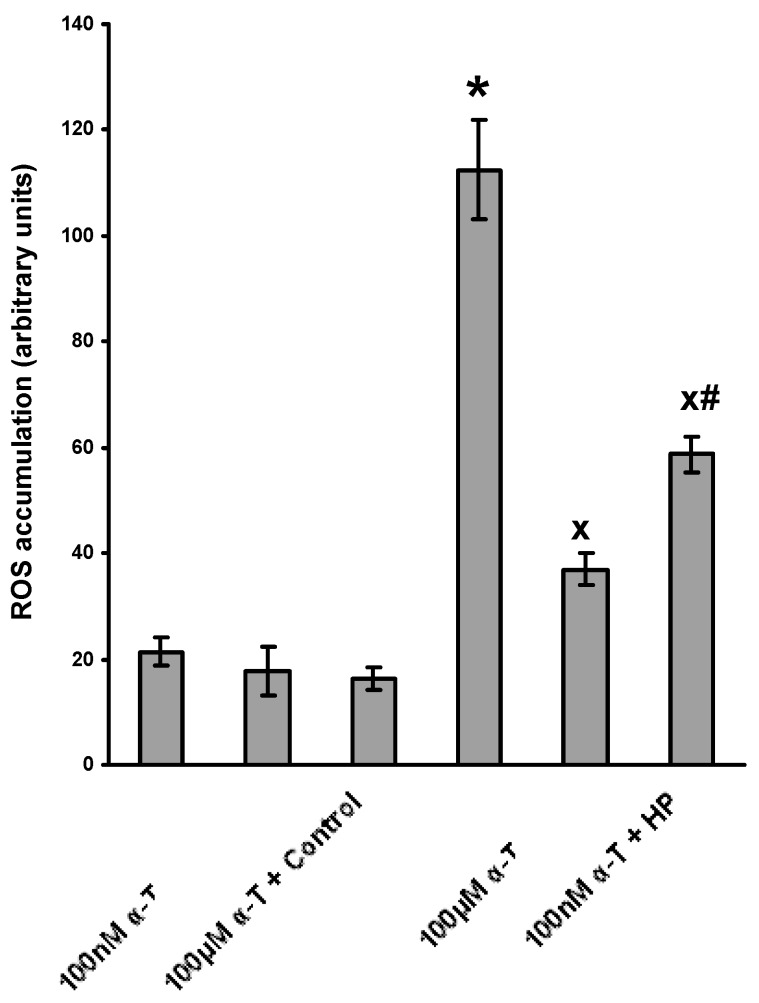
Shows that long preincubation with α-tocopherol (α-T) at nanomolar and micromolar concentrations diminished ROS accumulation in brain cortical neuron elicited by H_2_O_2_ to a great extent. The neurons were preincubated with α-T for 18 h. Then the fluorescent dye dichlorodihydrofluorescein diacetate was added to the incubation medium to a final concentration of 25 µM (see “Materials and Methods”). After 40 min incubation, the cells were exposed to 0.2 mM H_2_O_2_ for 4 h. H_2_O_2_ in the Figure is designated as HP. The data bars (from the left to the right) show: (1) control values; (2) control values after incubation of the neurons with 100 nM α-T; (3) control values after incubation of the neurons with 100 µM α-T; (4) ROS accumulation (arbitrary units) after neuron exposure to H_2_O_2_; (5) ROS accumulation after preincubation of the neurons with 100 µM α-T prior to the cell exposure to H_2_O_2_; (6) ROS accumulation after preincubation of the neurons with 100 nM α-T prior to the cell exposure to H_2_O_2_. The results of one typical experiment are shown as means ± SEM from 4–6 determinations in parallel samples. One-way ANOVA followed by Tukey’s test for multiple comparison was used to assess significance of the differences between various groups of data. The differences were found to be significant: *—compared to control values; ^x^—compared to the effect of H_2_O_2_ alone; ^#^—compared to the effect of 100 µM α-T (*p* < 0.01 in all cases).

The data showing that α-T diminishes the accumulation of ROS in brain cortical neurons are presented in Figure 2.

The data obtained provide evidence that the average inhibition of H_2_O_2_-initiated ROS accumulation by preincubation with 100 µM and 100 nM α-T for 18 h was 75.9% ± 3.1% and 54.8% ± 7.1%, respectively; the antioxidative effect of micromolar and nanomolar α-T was highly significant (*p* < 0.001 and *p* < 0.01, respectively), the effect of 100 µM α-T being more pronounced than the effect of 100 nM α-T under the experimental conditions used (*p* < 0.05, *n* = 4).

In another series of experiments we tried to study ROS accumulation in brain cortical neurons after their long preincubation with nanomolar and micromolar α-T prior to long (24 h) exposure to 0.2 mM H_2_O_2_. However, under such a long exposure to H_2_O_2_ ROS accumulation was not significant in a large part of the experiments, so we failed to obtain reliable results. It appears that exposure to 0.2 mM H_2_O_2_ for 24 h is the optimal time to measure the viability of neurons, but not to assess the initial disturbances of the metabolic processes caused by this prooxidant in the nerve cells.

If the neurons were preincubated with α-T for 1.5 h and then exposed to H_2_O_2_ for 2 h, 100 µM α-T was found to reduce ROS accumulation almost to the control levels, while 100 nM α-T inhibited H_2_O_2_-induced ROS accumulation by 47.4% ± 1.5% (*n* = 5).

We have previously [23] shown that the ability of 10 and 100 nM α-T to decrease ROS accumulation initiated in PC12 cells by H_2_O_2_ was diminished or became insignificant in the presence of MEK1/2/ERK1/2 and PI 3-kinase/Akt pathways inhibitors (SL327 and LY294002, respectively). However, these inhibitors did not change the decrease of ROS accumulation as a result of short preincubation with 100 µM α-T, apparently due to its scavenging effect [23].

### 2.5. α-T Increases the Basal Level of pAkt and pERK1/2 in Brain Cortical Neurons, but Does Not Change Total Akt and ERK1/2 Levels

**Figure 3:**
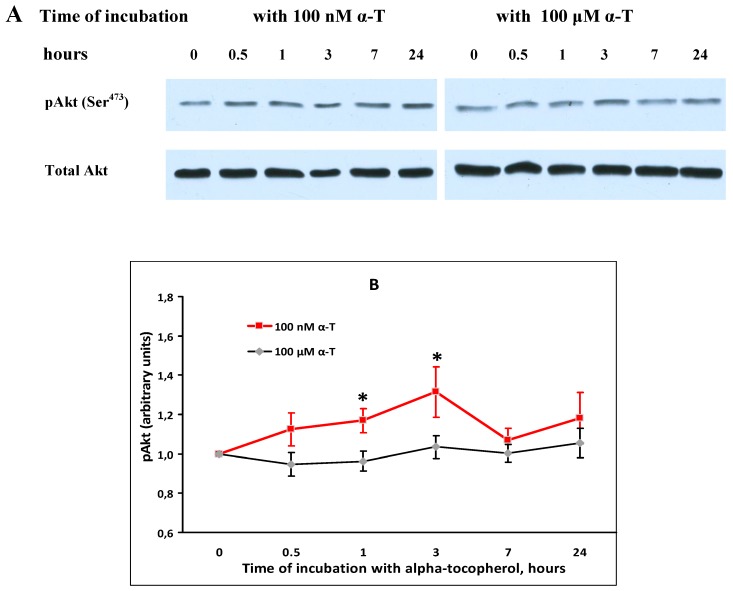
Shows the effect of incubation with 100 nM and 100 µM α-tocopherol (α-T) for 0.5, 1, 3, 7 and 24 h on the level of pAkt and total Akt in brain cortical neurons. Immunoblots obtained in one typical experiment from 6–7 experiments made are presented in (**A**). The data of 6–7 experiments made are presented as means ± SEM in (**B**). Red lines with squares show the effect of 100 nM α-T, black lines with rhombs the effect of 100 µM α-T. In this figure: *—the difference from the control level of pAkt is significant by paired Student’s *t* test after incubation with 100 nM α-T, *p* < 0.05.

The effect of α-T on basal Akt activity (pAkt level) and Akt level in cortical neurons was studied (Figure 3).

α-T (100 nM) was found to activate Akt (to increase pAkt level) in control brain cortical neurons 1 and 3 h after exposure of the cells to this antioxidant (Figure 3A). No change in pAkt level in these neurons was revealed as a result of their incubation with 100 μM α-T (Figure 3A). No change in total Akt level were evidenced after exposure of cortical neurons to 100 nM or 100 µM α-T, hence it had no influence on the expression of this protein kinase.

**Figure 4:**
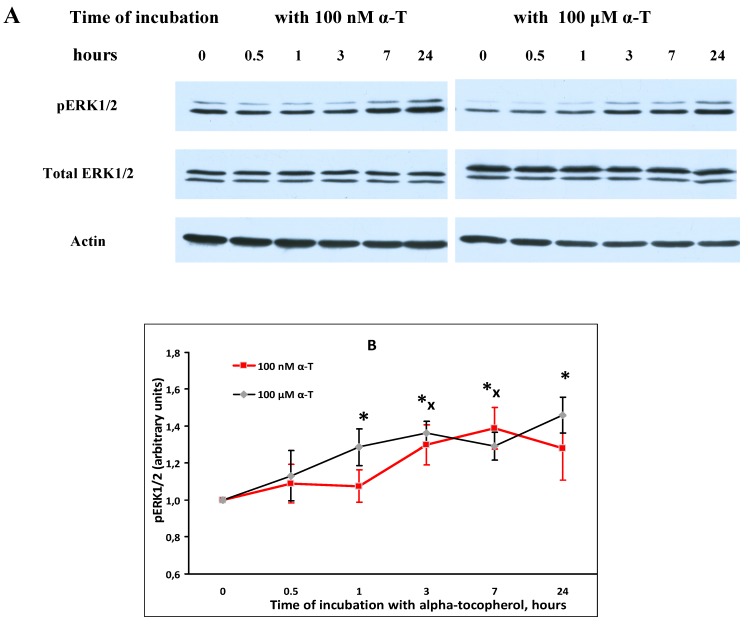
Shows that incubation of brain cortical neurons with 100 nM and 100 µM α-tocopherol (α-T) increased the level of pERK1/2 in these neurons. Immunoblots obtained in one typical experiment are presented in (**A**). The data are means ± SEM from 5–6 experiments in (**B**). Red lines with squares show the effect of 100 nM α-T, black lines with rhombs - the effect of 100 µM α-T. In this figure the difference is significant by paired Student’s *t*-test: *—between pERK1/2 level after exposure to 100 µM α-T and control pERK1/2 level, *p* < 0.05, **^x^**—between pERK1/2 level after exposure to 100 nM α-T and control pERK1/2 level, *p* < 0.05. The level of pERK1/2 significantly increased in brain cortical neurons after their exposure to 100 µM α-T for 1, 3, 7 and 24 h and to 100 nM α-T for 3 and 7 h. However, neither 100 nM, nor 100 µM α-T changed the total ERK1/2 level in brain cortical neurons, so it had no influence on the expression of this protein kinase.

The effect of α-T on basal ERK1/2 activity (pERK1/2 level) and total ERK1/2 level in brain cortical neurons was studied (Figure 4).

Numakawa and co-authors were the first to show that α-T (as well as γ-tocopherol) at nanomolar concentrations increases the basal activity of Akt and ERK1/2 in brain cortical neurons [10]. Our data are in agreement with their findings, but neither in this work nor in other publications the effect of α-T on activities of these protein kinases in neurons or cells of neuronal cell lines was studied under the conditions of oxidative stress.

### 2.6. While α-T Prevents Akt Inactivation Initiated by H_2_O_2_ in Brain Cortical Neurons, the Effect of 100 nM and 100 μM α-T Is Similar

**Figure 5:**
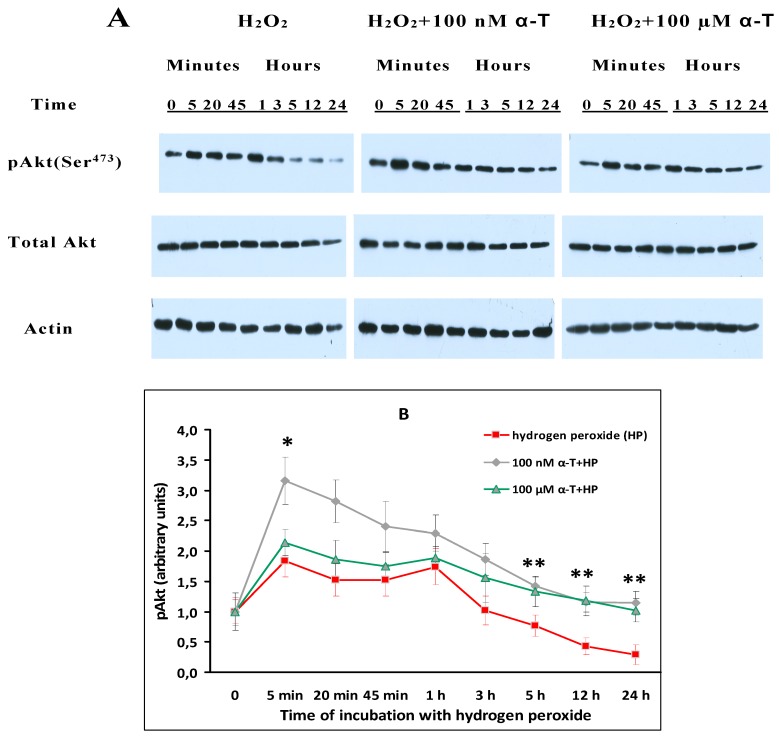
Shows the effect of preincubation of brain cortical neurons with 100 nM and 100 μM α-tocopherol (α-T) for 18 h prior to their exposure to 0.2 mM H_2_O_2_ for 24 h on pAkt and total Akt levels. Immunoblots obtained in one typical experiment are presented in (**A**). The results of 5–6 experiments are presented in (**B**) as means ± SEM. Red lines with squares show the effect of H_2_O_2_ alone, black lines with rhombs—effect of H_2_O_2_ after preincubation with 100 nM α-T, green lines with triangles—effect of H_2_O_2_ after preincubation with 100 μM α-T. In this figure: * and **—the differences are significant according to Student’s paired *t* test as compared to the level of pERK1/2 in brain cortical neurons exposed to H_2_O_2_ alone; *—the effect of preincubation with 100 nM α-T is significant, *p* < 0.05; **—the effect of preincubation with both 100 nM and 100 µM α-T is significant, *p* < 0.05.

The data presented in Figure 5 provide evidence that α-T prevents Akt inactivation initiated by H_2_O_2_ in cortical neurons.

The data obtained provide evidence that exposure of brain cortical neurons to 0.2 mM H_2_O_2_ results in inactivation of Akt. Thus, the activity of this protein kinase (measured as pAkt level) fell more than twice with respect to control values 12 h after the application of H_2_O_2_ and more than three times 24 h after the application of this prooxidant (Figure 5), the difference with the control level being significant (*p* < 0.01). Preincubation with 100 nM and 100 µM α-T markedly and significantly increased Akt activity in cortical neurons (Figure 5) 12 and 24 h after their exposure to prooxidant with respect to the effect of H_2_O_2_ alone. During the early stages (first 5 min),of exposure of cortical neurons to H_2_O_2_ 100 nM α-T increased the pAkt level as compared to the effect of H_2_O_2_ alone, whereas 100 µM α-T did not. Both H_2_O_2_ and α-T had no effect on the total Akt level (Figure 5A), which means that they did not change the expression of this enzyme. It is of interest that in contrast to brain cortical neurons, exposure of PC12 cells to 0.2 mM H_2_O_2_ for 24 h did not result in a pronounced inactivation of Akt [14].

The abrupt drop of Akt activity (Figure 5) revealed in our experiments as a result of cell exposure to H_2_O_2_ appears to be one of the reasons for neuron death. α-T prevents the inactivation of Akt under conditions of oxidative stress, but in presence of the PI 3-kinase/Akt signaling pathway inhibitor (LY294002) the protective effect of α-T was not observed (see Table 2). These data suggest that the ability of α-T to prevent inactivation of Akt in cortical neurons plays an important role in its protective effect against H_2_O_2_ toxicity.

It should be noted that activation of Akt by neurotrophins, flavonoids or other compounds usually leads to an increase of cells (including neurons) viability [24,25]. Conversely, inactivation of Akt results in cell damage and death (see, for example, [26]).

The activation of Akt may occur only after its binding to membrane phosphatidyl-inositol-3, 4, 5-phosphate formed by activated PI 3-kinase. The activation of PI 3-kinase takes place after a stimulus triggered by G protein coupled receptor or receptor tyrosine kinase. H_2_O_2_ was shown to activate Trk receptor tyrosine kinase [27]. The transient phosphorylation of Akt by H_2_O_2_ takes place downstream of activation of this protein kinase. Thus, the inhibitor of Trk tyrosine kinase (K252a) was shown to diminish Akt activity in PC12 cells exposed to H_2_O_2_ practically to the control level [14]. α-T activates Akt in the nerve cells [10,14], but has no effect on the activity of Trk tyrosine kinase. It is possible that α-T like glutaredoxin [28] changes the redox state of Akt preventing its oxidation and formation of disulfide bond between two cystein residues of these enzyme elicited by H_2_O_2_. Such changes of Akt structure induced by H_2_O_2_ facilitate association of Akt with protein phosphatase 2A, its dephosphorylation and subsequent degradation [28], but the presence of antioxidants like glutaredoxin [28] or α-T may prevent the effect of prooxidant. In our study, ROS formation in brain cortical neurons exposed to H_2_O_2_ was markedly diminished both by 100 µM, and by 100 nM α-T. Quite possible that it is a result of inhibition of one of the enzymes producing ROS by α-T [7].

### 2.7. While α-T Decreases the Time of Long Activation of ERK1/2 in Brain Cortical Neurons Initiated by H_2_O_2_, the Effect of 100 nM and 100 μM α-T Is Similar

**Figure 6:**
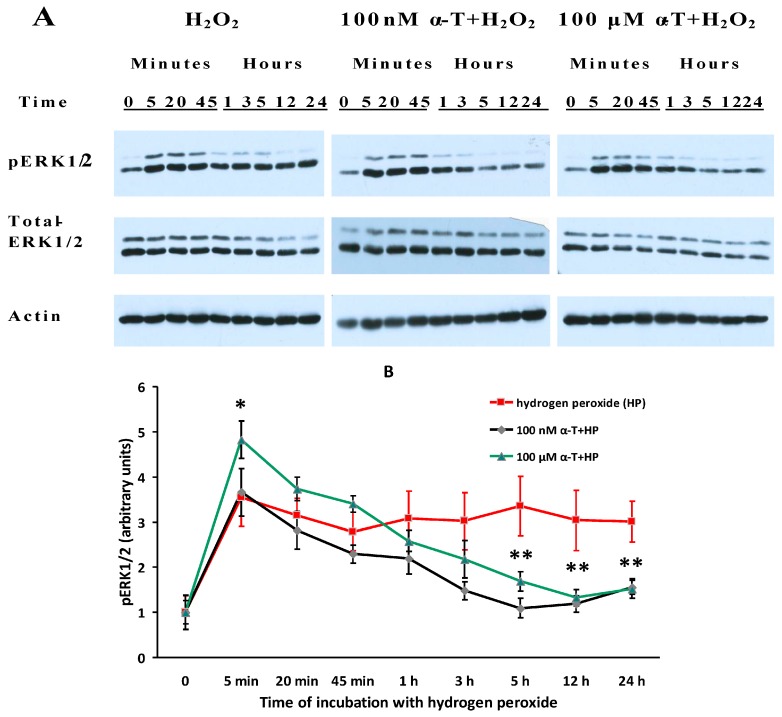
Shows the effect of preincubation of brain cortical neurons with 100 nM and 100 μM α-tocopherol (α-T) for 18 h prior to the cell exposure to 0.2 mM H_2_O_2_ for 24 h on pERK1/2 and total ERK1/2 levels. The immunoblots obtained in one typical experiment are presented in (**A**). The results of 5–6 experiments are presented in (**B**) as means ± SEM. Red lines with squares show the effect of H_2_O_2_ alone, black lines with rhombs—effect of H_2_O_2_ after preincubation with 100 nM α-T, green lines with triangles—effect of H_2_O_2_ after preincubation with 100 μM α-T. It is shown that 0.2 mM H_2_O_2_ activated ERK1/2 in brain cortical neurons (increased pERK1/2 level) 5 min after its application, then ERK1/2 remained at the same high level during 24 h of prooxidant action. However, if these neurons were preincubated with 100 nM and 100 μM α-T for 18 h and then exposed to 0.2 mM H_2_O_2_ for 24 h, the activity of ERK1/2 was not high, it was close to control values 12 and 24 h after exposure of the neurons to this prooxidant, the effect of preincubation with 100 nM and 100 µM α-T was significant. Preincubation with α-T (100 µM) caused a significant increase of the pERK1/2 level as compared to the effect of H_2_O_2_ alone at early stages of its action—5 min after cell exposure to H_2_O_2_, but 100 nM α-T did not exert such an effect. No change in the total ERK1/2 level was revealed as a result of the exposure of neurons to H_2_O_2_ alone or to H_2_O_2_ after preincubation with α-T, which means that the expression of this protein kinase was not changed. In this figure: * and **—the differences are significant according to Student’s paired *t* test as compared to the level of pERK1/2 in brain cortical neurons exposed to H_2_O_2_ alone; *—the effect of preincubation with 100 µM α-T is significant; *p* < 0.05, **—the effect of preincubation with both 100 nM and 100 µM α-T is significant, *p* < 0.05.

The data presented in Figure 6 provide evidence that α-T decreases the time of maximal activation of ERK1/2 initiated by H_2_O_2_ in brain cortical neurons.

The maximal level of pERK1/2 was reached within 5 min after neuron exposure to H_2_O_2_ and was then maintained for the whole 24 h observation period (Figure 6A,B). Preincubation with nanomolar and micromolar α-T prevented long and sustained activation of ERK1/2 initiated by H_2_O_2_ in brain cortical neurons. pERK1/2 levels were much lower in neurons preincubated with 100 nM and 100 µM α-T 5, 12 and 24 h after beginning to expose the cells to H_2_O_2_ (*p* < 0.05). In cells exposed both to α-T and H_2_O_2_, a peak instead of a plateau is seen in the histograms (Figure 6B). Neither H_2_O_2_ nor α-T altered the expression of ERK1/2.

The prolonged activation of ERK1/2 by H_2_O_2_ in brain cortical neurons observed in our study is in agreement with previous data showing the activation of this enzyme by ROS [29,30]. α-T decreased the time of maximal activation of ERK1/2 in PC12 cells as well [14], but the maximal activation of ERK1/2 by H_2_O_2_ in these cells lasted for a much shorter time than in cortical neurons. It should be noted that carnosine has a similar effect on ERK1/2 activity; carnosine was shown to protect cerebellar granule cells against oxidative stress and to decrease the time of ERK1/2 activation by a prooxidant [31]. α-T similar effect may be the result of a protein phosphatase activation, especially of 2A. The mechanism of protein phosphatase activation by α-T appears to be complicated, as it needs a long preincubation with it [7,8,32]. Both α-T and its derivative which does not have radical scavenging activity were shown to be able to inhibit ERK1/2 activity [33].

It is of interest to note that a short activation of ERK1/2 by neurotrophins, flavonoids or other compounds increases the viability of nerve cells [24,25], whereas long sustained activation of ERK1/2, in contrast, leads to nerve cell death. Thus, the exposure of cortical neurons to toxic zinc concentrations was shown to result in a sustained and excessive activation of Ras/MEK1/2/ERK1/2 pathway, mitochondrial dysfunction and neuronal death [34,35]. Glutathione depletion of cortical neurons and cells of HT22 neuroblastoma cell line leads to oxidative stress, sustained activation of ERK1/2 and, ultimately, the death of these cells [36]. When the effect of glutamate on the HT22 neuronal cell line was studied it was found that early activation of ERK1/2 increases cell viability, while conversely, a late activation leads to their death [36]. In vivo oxidative stress caused by ischemic damage is accompanied by sustained activation of ERK1/2 in the brain [37,38,39]. The administration of inhibitors of MEK1/2/ERK1/2 pathway (including SL327 used in our experiments) to animals subjected to brain ischemia and reperfusion (accompanied by activation of free radical reactions) leads to a pronounced decrease of neuronal death in damaged brain regions and to a marked improvement of the animal functional state [37,38,39].

In our study, 100 nM and 100 µM α-T was able to activate ERK1/2 in control cells and at the early stages of the H_2_O_2_ action, and to inhibit this protein kinase at the late stages of the H_2_O_2_ action. The ability of 100 nM and 100 µM α-T to inhibit the ERK1/2 activity at the late stages of prooxidant action was shown to be similar. In the presence of theMEK1/2 inhibitor (SL327), which prevents ERK1/2 activation, the rescue rates of α-T were to a great extent and significantly diminished (Table 2). The data obtained suggest that the ability of α-T at nanomolar and micromolar concentrations to increase the viability of brain cortical neurons depends on its modulation of ERK1/2 activity.

### 2.8. α-T at 100 μM and 100 nM Concentrations Diminishes the Activation of PKCδ Initiated by H_2_O_2_ in Brain Cortical Neurons

**Figure 7:**
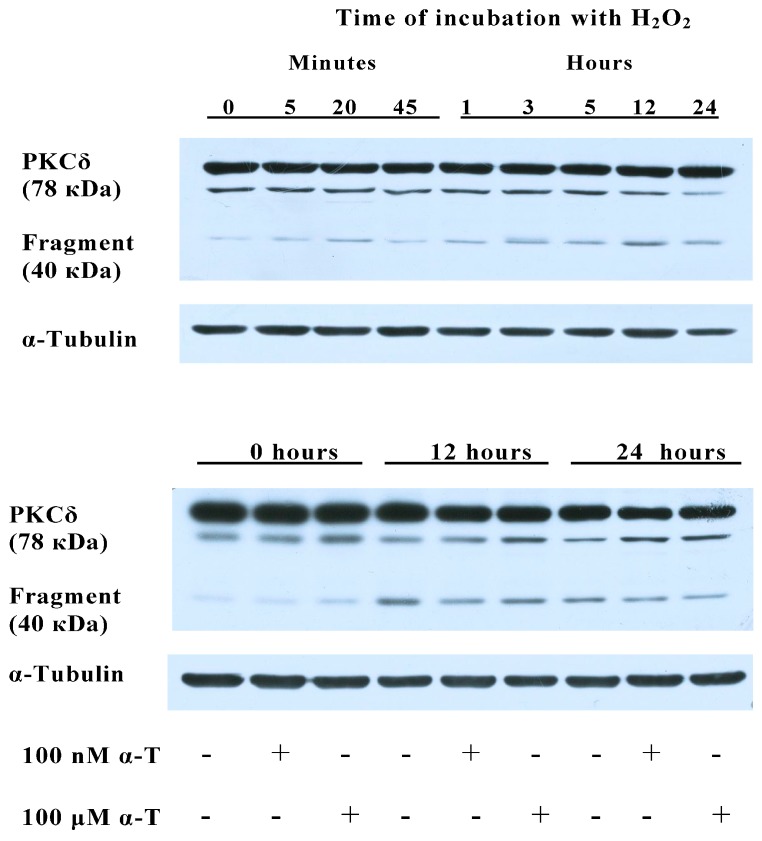
Shows the effect of H_2_O_2_ and preincubation with α-tocopherol (α-T) on the level of the active fragment of PKCδ with molecular mass 40 kDa and the level of total PKCδ in brain cortical neurons. These neurons were preincubated with 100 nM and 100 μM α-T (or without it) for 18 h and then exposed to 0.2 mM H_2_O_2_ for 24 h. Immunoblots obtained in one typical experiment (from 5 experiments made) show that H_2_O_2_ increased the level of catalytically active 40 kDa fragment of PKCδ in neurons up to 12 h after the beginning of their exposure to this prooxidant. It means that H_2_O_2_ activated PKCδ in brain cortical neurons. However, preincubation with 100 nM and 100 μM α-T diminished the increase of the level of 40 kDa fragment of PKCδ induced by H_2_O_2_. H_2_O_2_ and α-T had no effect on total PKCδ level (Figure 7), which means that they did not change the expression of this enzyme.

The activation of PKCδ may happen as a result of its phosphorylation or as a result of its proteolytic cleavage by caspase-3. In the latter case, a catalytically active 40–41 kDa fragment is formed as a result of the cleavage of 78–79 kDa PKCδ. Studies made using cells of the PC12 and N27 neuronal cell lines and smooth muscle cells provide evidence that proteolytic cleavage is the main way of PKCδ activation, leading to the apoptotic death of cells under conditions of oxidative stress initiated by H_2_O_2_ or by 6-hydroxydopamine extracellular auto-oxidation [40,41,42], whereas necrotic neuronal and muscle cell death is accompanied by increased phosphorylation of PKCδ, which results in the activation of this protein kinase [41,42]. We used long exposure to a relatively low H_2_O_2_ concentration (0.2 mM H_2_O_2_ for 24 h). In our previous work we have shown that, under conditions of oxidative stress initiated by such treatment, the apoptotic death of PC12 cells predominates [14]. That is why we studied the formation of the active 40 kDa fragment in order to assess the PKCδ activation in brain cortical neurons exposed to H_2_O_2_ (Figure 7).

Our data are in agreement with the data of Ferri and co-authors [43] who have shown that α-T inhibits PKCδ activity in the hippocampal dentate gyrus neurons in vivo. PKCδ activation was shown to increase the death of neurons in a number of studies [44,45,46]. Thus, it was shown [45] that the dopaminergic neurotoxicant 6-hydroxydopamine (6-OHDA) induced oxidative damage through the proteolytic activation of PKCδ in mesencephalic dopaminergic neuronal N27 cells. It is of interest that the activation of PKCδ was completely suppressed by treatment with a caspase-3-specific inhibitor. Expression of caspase-3 cleavage resistant mutant PKCδ (D327A) and kinase dead PKCδ (K376R) or siRNA-mediated knockdown of PKCδ protected against 6-OHDA-induced neuronal cell death [45]. In the works of Zhang and co-authors [44], it has been shown that the PKCδ isoform is an oxidative stress-sensitive kinase and a key mediator of apoptotic cell death in Parkinson’s disease models. Rottlerin was found to decrease PKCδ activity to a great extent in primary cultures of mesencephalic neurons. The neuroprotective effect of rottlerin was shown in both cell culture and preclinical animal models of Parkinson’s disease [44].

The generation of PKCδ catalytic fragment in cell nuclei by caspase-3 cleavage of PKCδ is a critical step leading to apoptotic cell death initiated by many apoptotic stimuli [47,48,49]. This 40–41 kDa fragment of PKCδ forms complexes with DNA protein kinases, phosphorylates and inactivates them, leads to chromatin condensation and nuclear fragmentation, to phosphorylation and activation of p73-beta (structural and functional homologue of the p53 tumor suppressor), to redistribution and activation of proapoptotic protein Bax that can directly induce cytochrome c release from the mitochondria, activates such enzymes as scramblases, responsible for phosphatidylserine translocation to outer leaflet of lipid bilayer, that leads to the apoptotic cell elimination [48,49,50]. It is of interest that the PKCδ catalytic fragment is not only a product of caspase-3 action on PKCδ, but is able to activate caspase-3 itself, enhancing the level of apoptosis [47,48]. Thus, PKCδ catalytic fragment has targets in various compartments of the cells, its numerous metabolic effects lead to cell apoptosis and death. In the present work we showed that neuron exposure to H_2_O_2_ results in the formation of catalytically active 40 kDa fragment of PKCδ. At the same time, preincubation of brain cortical neurons with 100 nM or 100 µMα-T markedly diminishes such activation. In the presence of the PKCδ inhibitor rottlerin, the protective effect of α-T against H_2_O_2_-induced death of cortical neurons was significantly lower than in its absence (see Table 2). All the above-mentioned data suggest that modulation of PKCδ activity by α-T makes a pronounced contribution to its protective effect against the toxic action of H_2_O_2_ on brain cortical neurons.

It should be noted that α-T inhibits the activity not only of PKCδ, but of other forms of this protein kinase as well, as has been shown in numerous studies. Thus, for example, the supplementation of pregnant rats with high doses of vitamin E or α-T was found to potentiate α-T incorporation in the hippocampus of their offsprings and to lead to a marked decrease of brain PKC phosphorylation throughout their postnatal maturation. Offsprings of α-T supplemented pregnant rats showed a pronounced reduction of long-term synaptic plasticity. The impairment of brain function was observed even in adulthood, thus, in adult rats a deficit in long-lasting spatial memory, which depends on the function of hippocampus, was shown [51]. The data obtained indicate [52] that gestational and neonatal exposure to supranutritional α-T intake can result in anatomical changes of the offspring hippocampus (in particular in an aberrant glia–synapse relationship) that last through adulthood.

In order to understand the physiological meaning of the results on the modulation of protein kinase activities by H_2_O_2_ and α-T in cultured neurons it is of importance to know if these results may be applied to brain in pathological conditions. The toxic effects of sustained ERK1/2 activation [37,38,39], of PKCδ activation by caspase-3 cleavage [44], of Akt oxidation and inactivation [28] are revealed in brain under pathological conditions concerned with activation of ROS formation. It gives additional interest to the data on the mechanism of nanomolar and micromolar α-T protective action in cultured nerve cells.

We studied the possible contribution of modulation of ERK1/2, Akt and PKCδ activity to the protective effect of α-T against H_2_O_2_-induced death of brain cortical neurons, but it is to be noted that the protective effect of α-T may depend also on modulation of other signaling pathways.

### 2.9. α-T at Micro- and Nanomolar Concentrations Prevents the Abrupt Decrease of Bcl-2 Level and the Marked Increase of the Bax/Bcl-2 Ratio Initiated by H_2_O in Brain Cortical Neurons

**Figure 8:**
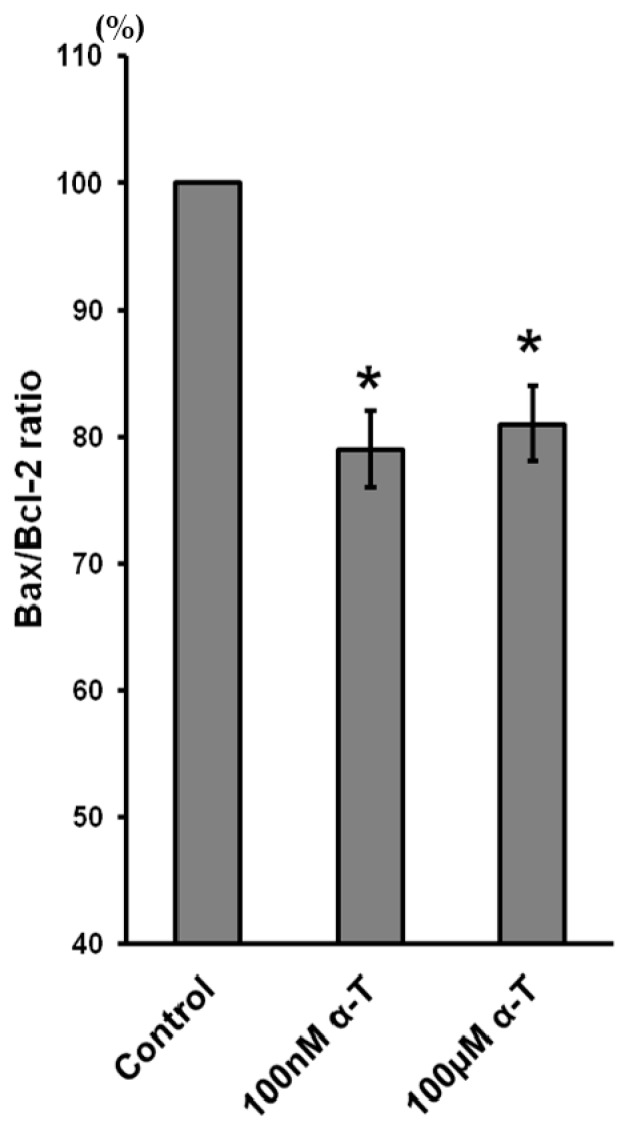
Shows the effect of incubation of brain cortical neurons with α-tocopherol (α-T) for 18 h on the basal Bax/Bcl-2 ratio. This ratio was taken as 100% in control brain cortical neurons. The data of five experiments made are presented as means ± SEM. α-T (100 nM and 100 µM) was shown to decrease the basal Bax/Bcl-2 ratio in brain cortical neurons. The diminution of this ratio was not pronounced, but it was significant. *—the difference is significant according to Student’s paired *t* test as compared to the Bax/Bcl-2 ratio in control cortical neurons, *p* < 0.02.

In order to elucidate the mechanism of the α-T protective effect under conditions of oxidative stress it is of importance not only to study its effect on the cell signaling pathways, but to obtain data on its ability to stabilize mitochondria, in particular to modulate the expression, level and activity of the pro- and antiapoptotic mitochondrial proteins. The effect of cortical neuron exposure to α-T on basal Bax/Bcl-2 ratio is shown in Figure 8.

**Figure 9:**
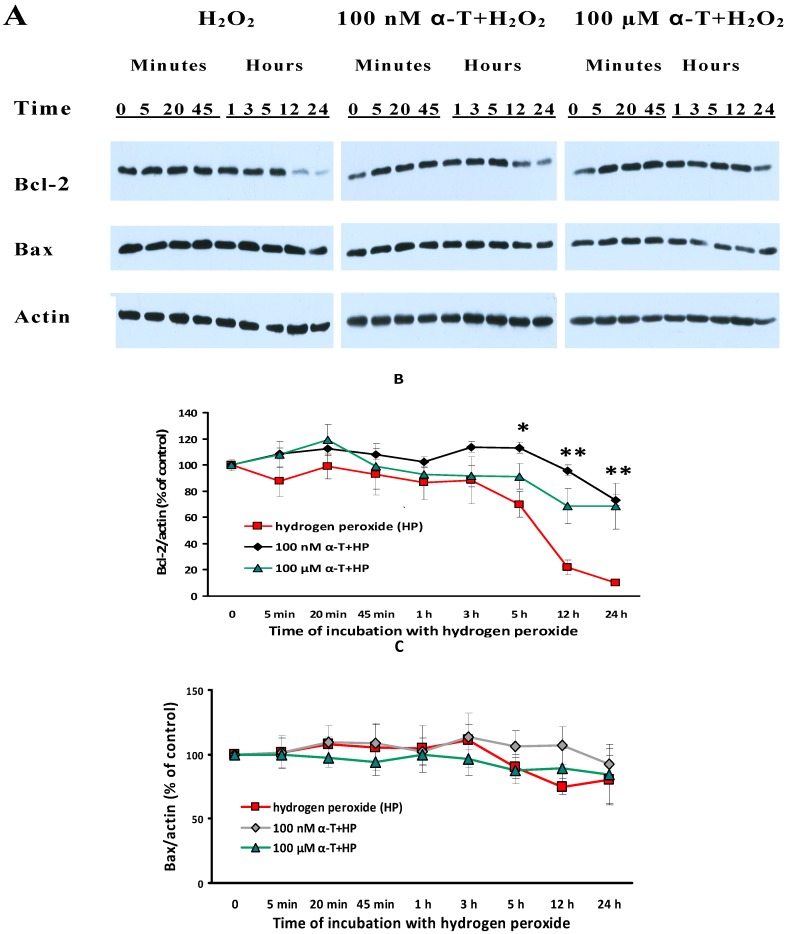
Shows the effect of exposure of cortical neurons to 0.2 mM H_2_O_2_ for 24 h and of preincubation with 100 nM and 100 μM α-tocopherol (α-T) for 18 h on the level of Bcl-2 and Bax in brain cortical neurons. The results of immunoblotting obtained in one typical experiment are shown in (**A**). The results of 6–7 experiments are shown in (**B**,**C**), respectively, as means ± SEM. Red lines with squares show the effect of H_2_O_2_ alone, black lines with rhombs—effect of H_2_O_2_ after preincubation with 100 nM α-T, green lines with triangles—effect of H_2_O_2_ after preincubation with 100 μM α-T. * and **—the differences are significant according to Student’s paired *t* test as compared to the level of Bcl-2 in brain cortical neurons exposed to H_2_O_2_ alone, * the effect of preincubation with 100 nM α-T is significant, * *p* < 0.05, ** the effect of preincubation with both 100 nM and 100 µM α-T is significant, *p* < 0.02.

But more important for us was to see the effect of nanomolar and micromolar α-T on the level of Bax and Bcl-2 under conditions of oxidative stress induced by H_2_O_2_ in brain cortical neurons. The experimental data obtained are presented in Figure 9.

It was found that H_2_O_2_ had practically no effect on the content of the antiapoptotic protein Bcl-2 in brain cortical neurons for the first 3 h after the application of H_2_O_2_. The small decrease in Bcl-2 level 5 h after exposure of the cells to H_2_O_2_ was found to be significant (*p* < 0.05). Then, an abrupt decrease in the level of this antiapoptotic protein started (Figure 9B). Thus, the Bcl-2 level 12 and 24 h after exposure of cortical neurons to H_2_O_2_ was much lower with respect to its initial level (0 point), this diminution being highly significant (*p* < 0.001). However if brain cortical neurons were preincubated with 100 nM or 100 μM α-T and then exposed to H_2_O_2_ the decrease in Bcl-2 content practically did not happen 12 and 24 h after cell exposure to this prooxidant. At these time intervals of exposure of the cells to H_2_O_2_, preincubation with 100 nM or 100 μM α-T significantly increased the Bcl-2 level in cortical neurons compared to the effect of H_2_O_2_ alone (Figure 9B).

The proapoptotic protein Bax did not show such a pronounced alteration of its level after brain cortical neuron exposure to H_2_O_2_ as Bcl-2. Preincubation with 100 nM or 100 μM α-T did not have any significant effect on Bax level (Figure 9C).

**Figure 10:**
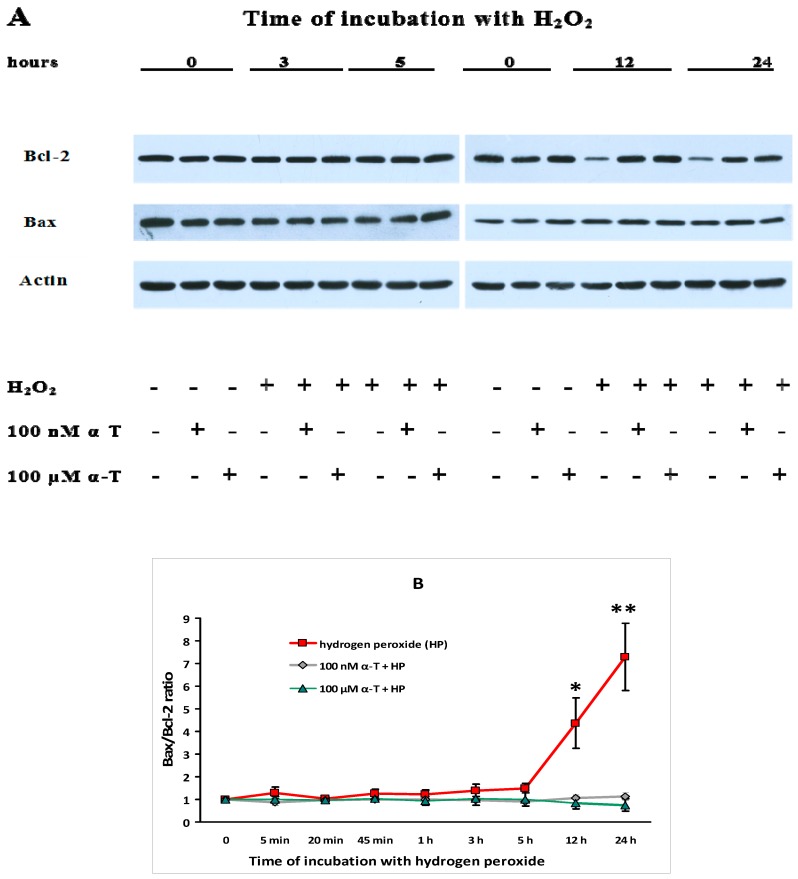
Shows the effect of H_2_O_2_ and preincubation with α-tocopherol (α-T) on the Bax/Bcl-2 ratio in brain cortical neurons. The levels of Bcl-2 and Bax 3, 5, 12 and 24 h after brain cortical neuron exposure to 0.2 mM H_2_O_2_ after preincubation for 18 h with 100 nM α-T and 100 μM α-T (or without it) are shown in (**A**). The results of five experiments on the Bax/Bcl-2 ratio in brain cortical neurons are presented as means ± SEM in (**B**). Red lines with squares show the effect of H_2_O_2_ alone, black lines with rhombs—effect of H_2_O_2_ after preincubation with 100 nM α-T, green lines with triangles—effect of H_2_O_2_ after preincubation with 100 μM α-T. * and **—the differences are significant according to Student’s paired *t* test as compared to the initial level of Bax/Bcl-2 ratio (0 point) and to the level of this ratio in brain cortical neurons exposed to H_2_O_2_ after preincubation with 100 nM and 100 μM α-T, * *p* < 0.05, ** *p* < 0.02. It means that the effect of preincubation with both 100 nM and 100 µM α-T is significant.

The data showing the effect of H_2_O_2_ and α-T on Bax/Bcl-2 ratio in brain cortical neurons are presented in Figure 10.

A pronounced and significant increase of the Bax/Bcl-2 ratio 12 and 24 h after exposure of cortical neurons to H_2_O_2_ alone appears to be mainly a result of the abrupt decrease of Bcl-2 level under conditions of oxidative stress. However, preincubation with nanomolar or micromolar α-T resulted in a pronounced increase of Bcl-2 level 12 and 24 h after brain neuron exposure to the prooxidant, as clearly seen in the immunoblots (Figure 10A). Our data showing that preincubation of neurons with α-T decreases Bax/Bcl-2 ratio (or increases “survival index”—Bcl-2/Bax ratio) in the neurons are in agreement with the data of Then and co-authors [53].

The long effect of 0.2 mM H_2_O_2_ on neurons or neuronal cell lines usually leads mainly to apoptotic cell death; thus it was shown in our study of the effect of H_2_O_2_ on PC12 cells [14], but necrotic death of a certain part of brain cortical neurons exposed to H_2_O_2_ cannot be excluded.

Proapoptotic mitochondrial proteins play an important role in the apoptotic death of various cells including neurons, while the increase of expression and level of antiapoptotic proteins, in contrast, decreases the apoptotic death rate (see, for example [54,55]). It is of interest that such proapoptotic proteins as Bax and Bak were at first considered to have no effect on the necrotic cell death, until data were obtained on their interaction with adenine nucleotide translocase which plays an important role in the permeability of the mitochondrial inner membrane and in mPTP function [56,57,58]. It was shown that Bax and Bak deletion decreases necrotic damage and death of cells in knockout mice with myocardial infarction [58], while the excessive expression of the antiapoptotic protein Bcl-2 may lead to the diminution of necrotic cell death [59]. It appears that mitochondrial pro- and antiapoptotic proteins like Bax, Bak, Bcl-2 and others determine both apoptotic and necrotic cell death.

In the literature there are data showing that the expression, level and activity of mitochondrial anti- and proapoptotic proteins may depend on the activity of Akt, ERK1/2 [25,60] and PKC [56,61]. The effect of activation of various forms of PKC may be opposite [61]. According to our data presented above the inactivation of Akt and the maintenance of maximal ERK1/2 activity are induced by H_2_O_2_ at approximately the same time or somewhat earlier than the great decrease of Bcl-2 level and the increase of Bax/Bcl-2 ratio in brain cortical neurons. At the same time, preincubation with α-T prevents both the alterations in the activity of ERK1/2 and Akt, and the decrease of the Bcl-2 level and increase of the Bax/Bcl-2 ratio in brain cortical neurons. It may be suggested that the normalization of Akt, ERK1/2 and PKCδ activity by α-T in brain cortical neurons exposed to H_2_O_2_ makes a great contribution to normalization of the Bax/Bcl-2 ratio in these cells. It is of interest that all these metabolic effects of α-T were very similar if it was used at micromolar or nanomolar concentrations.

## 3. Materials and Methods

### 3.1. Materials

α-T, H_2_O_2_, NADH, cytosine arabinoside were purchased in Sigma (Saint-Louis, MO, USA), K-252a, SL327, LY284002 and rottlerin were obtained from Calbiochem (San Diego, CA, USA), penicillin and streptomycin came from Serva (Heidelberg, Germany). The incubation media, Dulbecco’s modified Eagle Medium (DMEM) with l-glutamine, fetal calf blood serum were from Biolot Company (Saint-Petersburg, Russia). In the Immunoblotting section the information about various antibodies which were needed for experiments is given.

### 3.2. Brain Cortical Neurons in Culture

Brains of embryonic Wistar rat fetuses (day 17–18) were used to isolate cortical neurons and prepare primary cultures of cortical neurons by modified method of Dichter [62] as previously described [63]. Wistar rats were obtained from the Animal Facilities of I.M. Sechenov Institute of Evolutionary Physiology and Biochemistry of the Russian Academy of Sciences (IEPhB RAS, Saint-Petersburg, Russia). All procedures using animals were in accordance with the European Communities Council Directive of 24 November 1986, 86/609/EEC and were approved by the local Animal Care and Use Committee of IEPhB RAS. DMEM containing 10% fetal calf serum, 10% F12 medium, 2 mM glutamine and 20 mM Hepes was used as the complete incubation medium. Cells were seeded on poly-d-lysine coated 24-well plates at a density of 5 × 10^5^ cells per well. After 24 h, cytosine-arabinoside (1 μM) was added to culture for 24 h in order to minimize growth of glial cells. Culture medium was replaced every 3 days. Treatments were performed on the 5-th day in vitro. Preincubation of neurons with α-T was performed for 0.5 or 18 h in complete incubation medium prior to the exposure of cells to 0.2 mM H_2_O_2_ for 24 h in complete incubation medium. In some experiments neurons were preincubated in the presence of protein kinase inhibitors (SL327, LY294002 or rottlerin) for 0.5 h in complete incubation medium before the exposure of the cells to α-T.

### 3.3. Assessment of Cell Viability Using the Lactate Dehydrogenase (LDH) Method

LDH method was used to determine viability of immature brain cortical neurons. This method is based on evaluation of activity of LDH released to the incubation medium from the damaged cells. Before the aliquots were taken from the incubation medium the centrifugation of the samples was performed. In order to determine the activity of LDH in the samples the decrease of NADH level was measured in them. This reaction took place in the incubation medium containing 80 mM tris-HCl pH 7.2, 1.6 mM pyruvate, 0.2 mM NADH and 200 mM NaCl. In order to measure the decrease of NADH level the decrease of optical density of the samples was registered at 340 nm during approximately 5–6 min as previously described [64], M40 spectrophotometer (Karl-Zeisse, Germany) was used for this purpose. The total LDH activity of the samples was determined after the lysis of the neurons. It was performed in presence of 1% Triton X-100 at room temperature. The percent of LDH activity released to the incubation medium from the damaged cells to the total LDH activity in the sample was determined. If 100% of LDH activity is found in the incubation medium it means that all cells are dead.

In order to show the protective effect of α-T in various series of experiments its rescue rates were calculated. For this purpose the difference in the amount (activity) of LDH released from cortical neurons exposed to H_2_O_2_ in the absence and in the presence of of α-T was determined. Then the ratio of this difference to the increase of LDH amount released from neurons to the incubation medium in the presence of H_2_O_2_ alone (taken for 100%) was calculated. Such value corresponds to rescue rate of α-T against H_2_O_2_-induced neuron death. Thus, the rescue rate may be calculated in the following way: ([LDH release in H_2_O_2_−LDH release in H_2_O_2_ and α-T]/[LDH release in H_2_O_2_−LDH release in control]) × 100.

### 3.4. Determination of ROS Accumulation

Incubation with α-T was carried out as described in previous sections. The fluorescent dye dichlorodihydrofluorescein diacetate was added to the incubation medium to a final concentration of 25 µM. After 40 min incubation, the cells were exposed to 0.3 mM H_2_O_2_ for 4 h [65]. In order to remove dye excess the cells were washed with Hanks’ balanced salt solution. The fluorescence of the reaction product of ROS with dichlorodihydrofluorescein was determined using a Fluoroscan Ascent FL (Thermo Fisher Scientific, Vantaa, Finland) measuring the emission at λ = 523 nm after excitation at 485 nm. The ROS content was expressed in arbitrary units representing the intensity of the fluorescence of the reaction product.

### 3.5. Immunoblotting

Western-blot analysis was used to determine the expression and activity of ERK1/2, Akt and, expression of Bcl-2, Bax and PKCδ. Brain cortical neurons were exposed to α-T, H_2_O_2_ and other compounds like inhibitors (if needed), then they were washed twice using ice-cold PBS and scrapped in lysis buffer, which consisted of 50 mM Tris pH 8.0, 150 mM NaCl, 1% Triton X-100, 10 mM β-glycerophosphate Na, 10 mM NaF, 5 mM EDTA, 1 mM Na_3_VO_4_, 1 mM phenyl methyl sulfonyl fluoride (PMSF), and protease inhibitor cocktail (Roche, Mannheim, Germany). Complete lysis of cortical neurons was performed during 1 h on ice. To determine protein concentration in cell lysates the Lowry method with Folin and Ciocaltteu’s Phenol reagent was used, the protein determinations were performed in duplicate and bovine serum albumin was used as a standard. Protein-containing lysates were put in each lane in the equivalent amount (20–25 µg). Electrophoresis was performed in 10% sodium dodecyl sulfate—polyacrilamide gel. It was followed by transfer to pure nitrocellulose membranes (Schleicher & Schuell, Krackeler Scientific, Albany, NY, USA). The non-specific binding sites of the membranes were blocked with 20 mM Tris-HCl buffer (pH 7,6) containing 150 mM NaCl (TBS), 5% (*w/v*) skimmed milk and 0.1% Tween 20. The blots were then probed overnight with antibodies for pERK1 (pThr^202^/pTyr^204^) and pERK2 (pThr^185^/pTyr^187^) (1:2000, Sigma), pAkt (Ser^473^) (1:1000, Cell Signaling, Danvers, MA, USA), Bcl-2 (1:1000, Cell Signaling), Bax (1:1000, Cell Signaling) or PKCδ (C-17) (1:1000, Santa Cruz Biotechnology, Dallas, TX, USA). Then they were washes three times using 0.1% Tween 20 in TBS. Anti-mouse or anti-rabbit HRP-labeled secondary antibodies (Cell Signaling) were used. After the incubation with them for 1 h at the room temperature blots were developed, using for this purpose Enhanced chemiluminescence detection Wester blotting reagents (Amersham, GE Healthcare, Little Chalfont, UK). The data were normalized then. For this purpose membranes were incubated in the buffer, containing 65 mM Tris, pH 6.8, 2% SDS (*w/v*), and β-mercaptoethanol in order to strip antibodies previously used and re-probe with the antibodies for actin (1:100, Sigma), α-tubulin (1:2000), Akt (pan) (Cell Signaling) or total ERK1/2 (Cell Signaling). The quantification of optical densities of the positive bands of the scanned films was performed using NIH Image Analysis software version 1.43 (Bethesda, MD, USA).

### 3.6. Statistical Analysis

Data are presented as the means ± SEM. The significance of the differences between two groups of data was assessed by Student’s *t*-test and Student’s paired *t*-test. The statistical significance of differences between three or more groups of data was estimated using one-way analysis of variance (ANOVA) followed by Tukey’s post hoc multiple comparison test. Values of *p* < 0.05 were taken to be statistically significant.

## 4. Conclusions

Our work is one of the first attempts to study the mechanism of protective effect of α-T at nanomolar concentrations, which are its physiological concentrations in cerebrospinal fluid and brain extracellular space. The protective effect of α-T against the H_2_O_2_ toxic effect on the brain cortical neurons was found to be concentration-dependent in the nanomolar range (1 < 10 < 100 nM) if the preincubation with it was performed for 18 h. The maximal protection could be achieved by the preincubation for 18 h with 100 nM α-T in serum containing medium (complete incubation medium); a further increase of α-T concentration (1, 10 and 100 µM) did not result in a significant increase of the protective effect, but nanomolar α-T did not protect cortical neurons if preincubation was made for 0.5 h.

The possible contribution of modulation of ERK1/2, Akt and PKCδ activity by α-T to its protective effect against H_2_O_2_ toxicity was studied. Preincubation for 18 h with α-T at nanomolar and micromolar concentrations was found to prevent the inactivation of Akt and sustained activation of ERK1/2 maintained at a high level from 5 min to 24 h after exposure of the neurons to H_2_O_2_. Long preincubation with α-T diminished the activation of PKCδ elicited in cortical neurons by H_2_O_2_ alone. In the presence of inhibitors of MEK1/2/ERK1/2 or PI 3-kinase/Akt signaling pathways or of PKCδ the protective effect of α-T at nanomolar and micromolar concentrations was significantly diminished or disappeared. One of the ways by which modulation of activities of protein kinases ERK1/2, Akt and PKC may change the viability of neurons and other cells is their effect on the content of proapoptotic and antiapoptotic mitochondrial proteins. H_2_O_2_ was shown to cause an abrupt decrease of the level of Bcl-2 protein and a pronounced increase of the proapoptotic to antiapoptotic protein ratio (Bax/Bcl-2 ratio) in brain cortical neurons 12 and 24 h after the exposure of the cells to it, while after preincubation of neurons with α-T at nanomolar and micromolar concentrations Bcl-2 level was much higher and Bax/Bcl-2 ratio was much lower (close to control values) than in the case of neuron exposure to H_2_O_2_ alone.

α-T at concentrations of 100 nM and 100 μM was found to have an approximately similar metabolic effects on brain cortical neurons if preincubation with it was performed for 18 h. Thus, the ability of 100 nM and 100 μM α-T to modulate Akt, ERK1/2 and PKCδ activity and to prevent the changes of the Bax/Bcl-2 ratio under conditions of oxidative stress was found to be very similar. However, micromolar α-T diminished ROS formation induced by H_2_O_2_ to a higher extent than nanomolar α-T, but much shorter time of neuron exposure to H_2_O_2_ (4 h) was used in these experiments than in the case of determination of α-T effect on the viability of neurons (24 h).

It appears that the similar protective effect of nanomolar and micromolar α-T cannot be explained by the fact that nanomolar α-T may accumulate in neurons in the course of long preincubation. Thus, according to the data obtained by Saito and co-authors [13] the presence of 100 nM α-T in the incubation medium does not lead to any increase in the α-T content of brain cortical neurons after 24 h of incubation with this antioxidant, but the presence of 1 µM α-T in the incubation medium for 24 h leads to accumulation of approximately 250 pmol of α-T per mg of protein in brain cortical neurons. That is in contrast to α-tocotrienol which penetrates better to the cortical neurons and accumulates in them even if it is present in the incubation medium in nanomolar concentrations (100–250 nM) [13].

Taking into account the data on the similar protection of brain cortical neurons exposed to H_2_O_2_ by nanomolar and micromolar α-T obtained in the present study, a “more is better” approach to patients’ supplementation with vitamin E or α-T appears not to be reasonable. There are examples of unfavorable effects or possible unfavorable effects of vitamin E or α-T administration in high doses to humans and animals. They provide evidence that the mechanism of the protective action of α-T is quite complicated and not limited to scavenging activity only. As it was already mentioned, vitamin E administration in high doses was found to increase significantly the all-cause mortality in adult patients as well as for people in risk groups [1,2,3]. The possible mechanism of negative outcomes of supplementation of brain by α-T at high doses was shown in mice with acute ischemic brain stroke [4]. A poststroke increase in markers of oxidative injury and neurodegeneration and activation of microglia were shown to take place in the presence of elevated brain α-T. At supraphysiological level, α-T potentiated neuroinflammatory responces to active ischemic stroke [4]. Another example is a recommendation for an increased intake of the antioxidant α-T (vitamin E) by women in complicated pregnancies in order to prevent free radical damage to mother and fetus [52]. However, recent data showed that maternal vitamin E (α-T) supplementation to rats potentiated α-T incorporation in the brain (including hippocampus) of offsprings and led to a marked decrease of PKC phosphorylation, marked reduction of long-term synaptic plasticity in juvenile hippocampus and even to a deficit in long-lasting spatial memory in adulthood of offspring [51,52]. Such results raise concerns about the potential effects of increased α-T (vitamin E) intake by pregnant women on fetal brain development. All these data suggest that it is of importance to perform further studies of mechanism of α-T action on neurons and other brain cells at its physiological nanomolar concentrations present in brain extracellular space making its comparison with the mechanism of α-T action at much higher micromolar concentrations.

α-T belongs to the protectors that modulate signaling pathways and decrease the intensity of free radical reactions in neurons and other cells, like flavonoids, *N*-acetyl-l-carnosine, gangliosides and insulin. If the mechanism of the protective action of these natural compounds is better understood, there is a chance to reveal the combinations in which they additively or synergistically increase the protective effect of each other. Finally, the common use of some of these compounds may appear effective in preclinical and clinical trials as a remedy in neurodegenerative and brain ischemic diseases. It seems that such an approach might have an advantage compared to attempts to reveal the protective effect of α-T or vitamin E administration over a long time and in appreciable amounts to patients with various diseases and people in risk groups.

## Figures and Tables

**Table 1:** Shows the protective effect of preincubation with α-T for 18 h prior to brain cortical neuron exposure to 0.2 mM H_2_O_2_ for 24 h expressed as rescue rates of α-T. Cell viability was assessed by the LDH method. The data are means ± SEM from 7–9 experiments. The difference in the LDH activity released from cortical neurons exposed to H_2_O_2_ in the absence and presence of α-T was determined. The ratio of this difference to the increase of LDH activity released from neurons to the medium in the presence of H_2_O_2_ alone (taken as 100%) corresponded to the rescue rates of α-T against H_2_O_2_-induced cell death. The formula is ([LDH release in H_2_O_2_ − LDH release in H_2_O_2_ and α-T]/[LDH release in H_2_O_2_ − LDH release in control]) x 100. In this table: *—the protective effect of α-T is significant, *p* < 0.01; **^x^** and **^#^**—the differences are significant according to Student’s *t* test as compared to the effect of α-T at lower concentrations, **^x^** *p* < 0.02, **^#^** *p* < 0.01.

α-T Concentration	100 μM α-T	10 μM α-T	1 μM α-T	100 nM α-T	10 nM α-T	1 nM α-T
Rescue rates (%)	64.3 ± 7.2 *	67.4 ± 11.9 *	60.0 ± 11.4 *	52.5 ± 7.4 *^x^	27.3 ± 5.1 *^#^	5.1 ± 2.9

**Table 2:** Shows that the rescue rates of α-T against H_2_O_2_-induced brain cortical neuron death were significantly lower in the presence of an inhibitor of PI 3-kinase (LY294002), an inhibitor of MEK1/2 (SL327) and an inhibitor of PKCδ (rottlerin) than in their absence in the incubation medium. Cell viability was assessed by the LDH method. The data are means ± SEM from 5–6 experiments. Preincubation with protein kinase inhibitors was performed for 0.5 h, then α-T was added for 18 h before brain cortical neuron exposure to 0.2 mM H_2_O_2_ for 24 h. In this table: ** and *—the protective effect of α-T is significant, ** *p* < 0.02, * *p* < 0.05; **^x^** and ^#^—the differences are significant as compared to the effect of α-T in the absence of inhibitors by paired Student’s *t*-test, **^x^** *p* < 0.02, **^#^** *p* < 0.05.

Sample	Rescue Rates of α-T, %	Sample	Rescue Rates of α-T, %
100 nM α-T	52.4 ± 13.1 **	100 μM α-T	63.35 ± 12.6 **
100 nM α-T + 10 µM SL327	31.35 ± 11.1 *^,#^	100 μM α-T + 10 μM SL327	36.9 ± 12.3 *
100 nM α-T + 50 µM LY294002	10.1 ± 4.6 ^x^	100 μM α-T + 50 μM LY294002	27.6 ± 14.7 ^#^
100 nM α-T	50.0 ± 7.5 **	100 μM α-T	52.2 ± 6.4 **
100 nM α-T + 5 µM rottlerin	20.9 ± 4.6 **^,x^	100 μM α-T + 5 μM rottlerin	33.6 ± 4.8 **^,x^

## Abbreviations

α-T	α-tocopherol
ERK1/2	extracellular signal-regulated protein kinase
PI 3-kinase	phosphatidylinositol 3-kinase
Akt	protein kinase B
PKC	protein kinase C
ROS	reactive oxygen species
